# Impact of menopausal symptoms on work and careers: a cross-sectional study

**DOI:** 10.1093/occmed/kqad078

**Published:** 2023-08-05

**Authors:** M T O’Neill, V Jones, A Reid

**Affiliations:** Occupational Health & Wellbeing Department, Tallaght University Hospital, Dublin D24NR0A, Ireland; Occupational Health & Wellbeing Department, Tallaght University Hospital, Dublin D24NR0A, Ireland; Occupational Health & Wellbeing Department, Tallaght University Hospital, Dublin D24NR0A, Ireland

## Abstract

**Background:**

Women over 50 years are one of the fastest-growing employment groups. Menopausal symptoms can adversely impact quality of life, work performance and attendance; however, few studies look at the impact of individual menopausal symptoms on work and career development.

**Aims:**

To measure the prevalence of menopausal symptoms in employees in a healthcare setting, to assess the impact of individual symptoms on work, attendance and career development and to explore perceptions about workplace supports.

**Methods:**

In this cross-sectional study of Irish hospital workers, menopausal employees were asked about the frequency of 10 menopausal symptoms and the extent to which each symptom impacted them at work. Impacts on performance, attendance and career development were assessed, along with the benefits of workplace support.

**Results:**

Responses from 407 women showed that the most common menopausal symptoms affecting employees greater than 50% of the time while at work were fatigue (54%), difficulty sleeping (47%), poor concentration (44%) and poor memory (40%). Work performance was impacted for 65% of respondents and 18% had taken sick leave. There was a significant association between symptom severity at work and reduced work performance, career development decisions and attendance. Manager awareness about menopause (29%) and flexible working times (29%) were selected as the most important workplace supports.

**Conclusions:**

Female employees are negatively impacted by menopausal symptoms while at work, particularly by psychological and neurocognitive symptoms which were associated with reduced work performance, attendance and career decisions. Manager awareness and flexible schedules were considered the most beneficial workplace supports.

## Teaser

Menopausal symptoms can impact occupational well-being, but few studies look at the impact of individual symptoms on work and career development. In this cross-sectional study of hospital workers, the most common menopausal symptoms affecting employees at work were fatigue, difficulty sleeping, poor concentration and memory. A significant association was found between symptoms and work performance, attendance, and career development. Manager awareness and flexible schedules ranked highly as workplace supports and should be prioritized by employers.

**Key learning points**

**What is already known about this subject**

- Menopausal symptoms can adversely affect quality of life, work performance and attendance.
- Most workers in the health and social care setting are female, a significant proportion of whom are over 50 years of age.
- Studies in menopause at work focus on the frequency of menopausal symptoms in general; however, few studies look at the impact of individual menopausal symptoms on work, attendance and career development.

**What this study adds**

- This study demonstrated that fatigue, difficulty sleeping, poor concentration and poor memory were the menopausal symptoms that impacted employees most at work.
- A significant association was found between symptoms which impacted upon work 50–100% of the time and work performance, career decisions, and attendance.
- Manager awareness about menopause and flexible work schedules were considered the most beneficial workplace supports.

**What impact this may have on practice or policy**

- These findings highlight that psychological and neurocognitive symptoms associated with menopause also need to be addressed in the workplace, in addition to vasomotor symptoms.
- Manager training about menopause and the option of flexible work schedules in certain circumstances should be prioritized in employers’ menopause-at-work policies.

## Introduction

Women over 50 years are one of the fastest-growing employment groups, with employment rates in those aged 50–64 years ranging from 55% to 67% across Europe, Australia [1,2] and the USA [3]. Most women in this age cohort will experience menopause, which occurs at a median age of 51 years (interquartile range 46–53 years) [4]; however, the menopausal transition period begins on average 4 years before this [5] and can last for between 4 and 8 years [6]. In addition to menstrual irregularities, menopause is associated with a range of symptoms including hot flushes, night sweats, sleep disturbances, mood changes, musculoskeletal pain [7] and subjective cognitive symptoms affecting memory such as forgetfulness and impaired recall [8], all of which can affect physical, psychological and occupational well-being [9].

Menopausal symptoms have been reported to adversely affect employees impacting work performance and reducing work satisfaction [9]. In addition to the effects of menopausal symptoms on individual occupational well-being, women’s participation in the workplace may be impacted resulting in direct and indirect costs to the employer and state [10,11]. As the life expectancy and working age of women increase, many can expect to spend more than one-third of their lives in menopause, with a significant proportion of this in employment.

Over the last decade, there has been an increase in awareness among employers of menopause as a potential occupational health concern and of the need for appropriate workplace support [12–14]. However, despite the increased research into menopause at work, few studies investigate the extent of the impact of individual menopausal symptoms on work and career development for employees. This is particularly relevant in female-dominant workforces. In the European Union, 75% of those working in the health and social care sector are women [15] and, in the UK, this sector is the largest employer of female workers, with 25% of those aged 50–64 years and 22% of those aged 35–49 years working in health and social care [16]. Studies conducted on healthcare workers often assess the frequency of menopausal symptoms [17] rather than the impact of specific symptoms on work or focus on outcomes such as quality of life [18] or coping strategies [19], rather than effects on careers and occupational well-being. The aim of this study, therefore, was to determine the frequency of specific menopausal symptoms at work in a predominantly female healthcare setting and to assess the impact of symptoms on attendance, work performance and career development. Perceptions about the benefits of various workplace supports were also sought to further inform the most appropriate workplace accommodations for menopausal workers.

## Methods

This was a cross-sectional study of female employees in a large Irish teaching hospital who were either experiencing menopausal symptoms at the time of the survey or who had already gone through menopause. The employees worked in various hospital departments and encompassed both clinical and non-clinical roles. Participation was voluntary and the questionnaire was accompanied by a participant information leaflet. All responses were anonymous and confidential.

The survey was administered using SurveyMonkey® and the link was distributed via email to all female employees in the hospital on two separate occasions. It was also promoted in the staff newsletter and on the hospital’s electronic noticeboard. Information posters with survey Quick Response codes were placed throughout the hospital along with hard copies of the questionnaire. Departments where employees did not have access to computers were identified and paper copies of the survey were distributed to those employees, in addition to off-site satellite units.

The questionnaire collected demographic information including age, area of work and hours worked, as well as information regarding the age of onset of menopausal symptoms, frequency and duration of each symptom and resources and treatments accessed. Participants were asked to rate the extent to which 10 menopausal symptoms affected them while at work with the options being; not impacted at all, impacted less than 50% of the time, impacted greater than 50% of the time or impacted all of the time. The 10 symptoms were hot flushes, night sweats, difficulty sleeping, fatigue, poor concentration, poor memory, mood changes or low mood, increased anxiety, muscle and joint pain, and headaches. Sick leave taken due to symptoms and the overall impact of menopause on work performance, career development, choice of roles and ability to work night shifts were also recorded. Respondents were asked if they had discussed symptoms with managers and about perceived manager support around menopause. They were also invited to rank various workplace supports. The remaining questions covered knowledge of menopause and the provision of information about menopause in the workplace. Free text was also provided for further comments.

The data are coded and statistically analysed using IBM® SPSS® Statistics V26. Descriptive analyses in the form of frequencies and percentage distributions were calculated for respondents overall. Chi-square goodness-of-fit tests were run for categorical variables to determine if the distribution of variables among respondents was like that within the hospital female workforce. Differences between groups were assessed using a chi-squared test of independence or Fisher’s exact test where cell sizes were less than 5 for categorical data. Mann–Whitney U-test or Kruskal–Wallis test was used for ordinal variables and the only continuous variable, hours worked per week, as these data were not normally distributed as per the Shapiro–Wilks test (*P *< 0.05). Two-tailed statistical significance was set a priori as P < 0.05. Ethical approval for the study was granted by the SJH/TUH Joint Research Ethics Committee.

## Results

In total, 407 employees completed the questionnaire, of whom 339 were aged 45–64 years thereby giving a response rate of 35% for all female workers aged 45–64 years (*n* = 956). With a total workforce of 4011, 76% of whom are female, the results represent 10% of all employees. The baseline characteristics are summarized in Table 1. Most respondents (58%) were aged 45–54 years and 25% were aged 55–64 years, representative of the distribution of female hospital employees aged between 45 and 64 years (*χ*^2^(1) = 1.47, *P* = 0.22). Areas of work included nursing (39%), management/administration (37%), and health and social care professionals (13%). The majority had clinical roles (60%) which was representative of female hospital employees (*χ*^2^(1) = 2.92, *P* = 0.08). Two-thirds worked 35 h or more per week (mean 32.2 h).

**Table 1.** Baseline characteristics of survey participants

Baseline characteristics of survey participants (*n* = 407)	
Age range (years)	
25–34	5 (1)
35–44	61 (15)
45–54	236 (58)
55–64	103 (25)
65–74	2 (1)
Area of work	
Nursing	159 (39)
Management and Admin	149 (37)
Health and Social Care Professionals (HSCP)	52 (13)
Other Patient Care^a^	21 (5)
Medical and Dental	18 (4)
General Support^a^	8 (2)
Hours worked per week	
Hours, mean ± SD	32.2 ± 7.1
Working ≥ 35 h per week	272 (67)
Age at onset symptoms (years)	
<35	12 (3)
35–40	40 (10)
41–45	141 (35)
46–52	175 (43)
53–55	27 (6)
>56	3 (1)
No symptoms	9 (2)
Duration of symptoms (*n* = 398)	
Symptoms still present	296 (74)
<1 year	9 (2)
1–3 years	27 (7)
4–7 years	32 (8)
8–10 years	15 (4)
>10 years	19 (5)
Resources/treatments used	
GP	247 (61)
Gynaecologist/menopause specialist	94 (23)
Websites/social media	162 (40)
Occupational Health and Wellbeing Department	19 (5)
Hormone replacement therapy	123 (30)
Antidepressants	46 (11)
Counselling	38 (9)
Alternative/complementary therapies	124 (30)

Results are presented as *n* (%).

^a^Other patient care are primarily healthcare assistants and general support includes catering, portering, technical services, hospital sterile services, lab aides and radiographer assistants.

Symptom onset commonly occurred between 46 and 52 years (43%), followed by 41–45 years (35%). Of those who were symptomatic (*n* = 398), almost three-quarters (74%) reported current symptoms and in the remainder; 8% had symptoms lasting 4–7 years, 2% had symptoms lasting under a year and 2% remained asymptomatic.

**Figure 1 .**
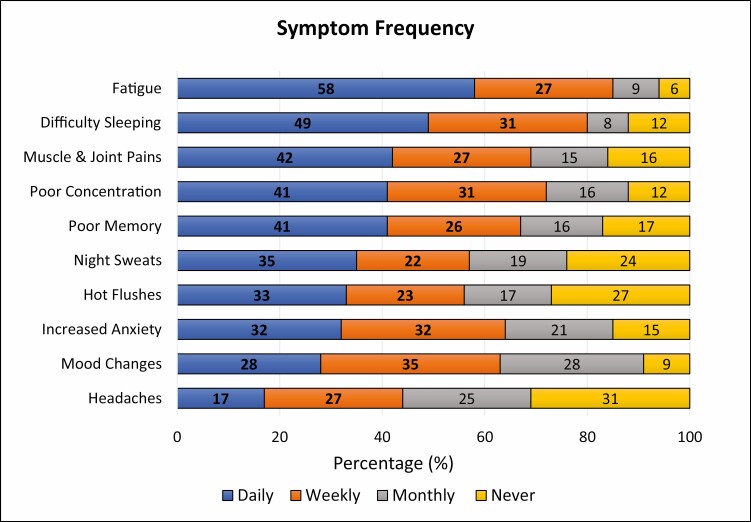
Frequency of menopausal symptoms.

Symptoms with the highest daily frequency were fatigue (58%), difficulty sleeping (49%), muscle/joint pain (42%), poor concentration (41%) and poor memory (41%) (Figure 1). Night sweats and hot flushes had a daily frequency of 35% and 33%, respectively. When daily and weekly symptoms were combined, fatigue (85%), difficulty sleeping (80%) and poor concentration (72%) again occurred most frequently. Night sweats and hot flushes had a combined daily and weekly frequency of 57% and 56%, respectively. Symptom frequency varied with age; hot flushes, night sweats, difficulty sleeping and muscle/joint pain were greater in those aged 45–64 years compared to those aged 35–44 years (*χ*^2^(2) = 24.7, *P* < 0.0005, *χ*^2^(2) = 18.5, *P* < 0.0005*, χ*^2^(2) = 13.8, *P* = 0.001, *χ*^2^(2) = 11.7, *P* = 0.003). Low mood was greater in those aged 45–54 years compared to 35–44 years (*χ*^2^(2) = 12.5, *P* = 0.002). No association was seen between symptom frequency and area of work.

**Figure 2 .**
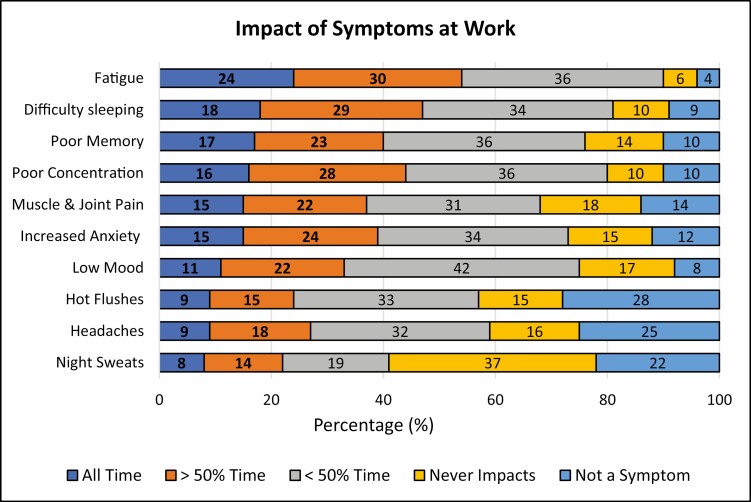
Percentage of time impacted by menopausal symptoms at work.

The most common symptoms affecting employees greater than 50% of the time while at work were fatigue (54%), difficulty sleeping (47%), poor concentration (44%) and poor memory (40%) (Figure 2). Hot flushes and night sweats were associated with a greater impact at work in employees aged 55–64 years than those aged 35–44 years (*χ*^2^(4) = 20.3, *P* < 0.0005, *χ*^2^(4) = 13.2, *P* = 0.01). Headaches impacted those working in management/administration and general support more than in other areas. No association was seen between the impact of other menopausal symptoms and area of work or number of hours worked.

**Figure 3 .**
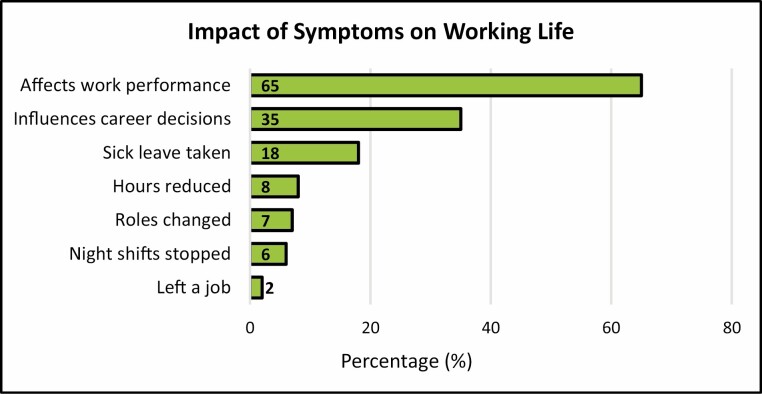
Impact of menopausal symptoms on working life.

When evaluating the impact of menopausal symptoms on working life, 65% stated that symptoms had affected their work performance, 35% reported career development decisions were influenced and 18% had taken sick leave. In addition, 8% of respondents reduced their hours, 7% changed roles, 6% stopped night shifts and 2% left a job due to symptoms (Figure 3). A significant association was found between all symptoms which impacted upon work 50–100% of the time and performance at work, career decisions and sick leave taken, apart from night sweats which were not associated with sick leave and headaches which were not associated with career decisions (Table 1, available as Supplementary data at *Occupational Medicine* Online). In addition, there was a significant association between those impacted by hot flushes, night sweats, fatigue or muscle pain at work, and changing roles, with the latter two symptoms also associated with stopping night shifts. Mood changes and anxiety at work were associated with reducing hours. Overall, no symptom was significantly associated with leaving a job.

In relation to absenteeism, 76% reported no absence due to symptoms, 13% missed 1–3 days, 6% missed 4–7 days, 1% missed 8–14 days and 4% missed more than 14 days annually. Attendance rates were similar across ages, areas of work, clinical or non-clinical roles, and between part-time and full-time employees. A significant association was seen between those who missed more than 7 days annually and all symptoms apart from headaches and joint pain (Table 1, available as Supplementary data at *Occupational Medicine* Online).

**Figure 4 .**
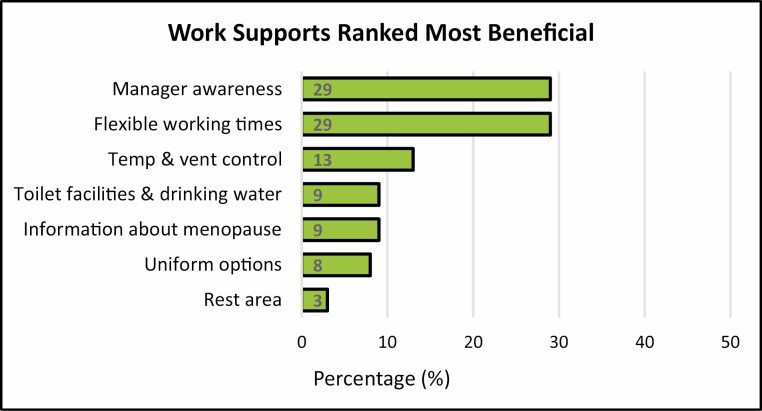
Work supports ranked most beneficial to assist with menopausal symptoms.

When ranking workplace supports, 29% selected manager awareness about menopause as the most important, followed closely by flexible working times (Figure 4). Temperature and ventilation control (13%), accessible toilet facilities and drinking water (9%), information about menopause (9%), and lightweight uniforms (8%) followed. The availability of a rest area was ranked least important (3%). There was no association between the ranking of workplace supports and age, area of work, or clinical and non-clinical roles. A significant association was found between those affected 50–100% of the time by hot flushes and temperature and ventilation control (*χ*^2^(6) = 23.0, *p* = 0.001).

When asked about discussing menopausal symptoms affecting them at work with their manager, 50% did not feel it was necessary, 13% had already discussed them, 6% hadn’t yet but were planning to and 31% would like to but didn’t feel comfortable doing so. There was no association between age or area of work and discussion of symptoms with management. Over half of the respondents (57%) disagreed or strongly disagreed with the statement that there was good awareness and support for employees affected with menopausal symptoms among management. Just 10% agreed or strongly agreed and 33% neither agreed nor disagreed. No association was found between opinion about manager support and age, area of work or clinical and non-clinical roles.

The most accessed resources were general practitioners (GPs) (61%), websites and social media (40%) and specialists (23%) with 5% attending Occupational Health (Table 1). Treatments included hormone replacement therapy (HRT) (30%), alternative/complementary treatments (30%) and antidepressants (11%). GP attendance and HRT use were significantly associated with those impacted by symptoms more than 50% of the time at work. Antidepressant use was significantly associated with all neurocognitive symptoms and low mood and anxiety were also associated with counselling. Most respondents (60%) rated their menopause knowledge as good or very good, with just 7% rating it as poor. No association was found between menopause knowledge and area of work or clinical and non-clinical roles.

Further comments were provided by 84 respondents (21%) addressing a range of topics. Additional symptoms were reported including menstrual flooding, urinary incontinence and isolation along with the impact of surgical menopause and premature ovarian insufficiency. The importance of workplace supports was frequently raised; particularly managerial support, information about menopause and enhanced flexibility including reduced hours, fewer night shifts, home-working and dedicated leave. Support received from Occupational Health was mentioned. Many participants complimented the study for highlighting menopausal symptoms in the workplace, particularly in a female-dominated environment.

## Discussion

This study of over 400 menopausal employees details the impact of menopausal symptoms upon work and the implications for performance, attendance and career decisions. The adverse effect of symptoms is consistent with other research [12,13,20,21]; however, this study provides further clarity on the extent to which individual menopausal symptoms impact upon work. Interestingly, neurocognitive and psychological symptoms predominated while vasomotor symptoms, more traditionally associated with menopause, impacted to a lesser extent while at work. Findings from previous studies vary when reporting those menopausal symptoms impacting most upon employee well-being. Similar to the findings in this study, Griffiths *et al*. [12] reported poor concentration, tiredness, poor memory and low mood to be most problematic for work, and Geukes *et al.* [21] found psychological and somatic symptoms to be more significant predictors of workability than vasomotor symptoms. In contrast, Williams *et al.* [22] reported that women withsevere vasomotor symptoms were nearly three times as likely to have a negative impact on work life compared to those with mild or moderate vasomotor symptoms, and Gartoulla *et al*. [23] found that vasomotor symptoms were associated with a greater likelihood of poor–moderate workability.

Presenteeism was a common theme, with almost two-thirds of respondents reporting reduced work performance associated with menopausal symptoms. This is greater than that reported by Griffiths *et al*. [12] where 39% agreed or strongly agreed that their performance had been negatively affected by menopausal symptoms. Studies, when looking at the impact of menopause on work, often report on reduced workability [13,21,23] and productivity [20]; however, few examine the longer-term implications of symptoms on employees’ working lives and careers. In this study, symptoms impacting work were significantly associated with career development, with over one-third of workers stating that menopausal symptoms influenced career decisions. Consistent with prior research [10,11,13,23], attendance was also adversely affected by menopausal symptoms as evidenced by the association observed between symptom severity and sick leave. Interestingly, occupation or number of hours worked was not associated with the impact of symptoms at work or absenteeism. The Health and Employment after Fifty Study [24] reported similar findings with no difference by type of job, physical work characteristics or night/shift work and difficulty with menopausal symptoms at work. Of note, fatigue, the symptom that impacts workers to the greatest extent, in addition to being associated with reduced performance at work and reduced attendance, was also significantly associated with changing roles and stopping night shifts. Fatigue and difficulty sleeping are commonly cited as impacting menopausal employees at work, suggesting that fatigue may adversely affect functional performance [12,25,26].

There has been increased awareness among employers in recent years of the need to support employees experiencing menopausal symptoms in the workplace [9,14]. In keeping with other studies [12], manager awareness and flexible work schedules were selected as the most beneficial workplace supports, ranking above temperature and ventilation control, the provision of information and lightweight uniforms. Recent recommendations published regarding menopause in the workplace prioritize manager training about menopause to enable them to support employees, implement practical workplace adjustments and signpost to additional supports [14,27,28]. Despite this, over half of respondents did not agree that manager support and awareness were present and almost one-third stated that they would like to discuss their symptoms with their manager but felt uncomfortable doing so.

The study has several limitations. As with all cross-sectional studies, it does not allow causal inferences to be made between variables and there may be other unmeasured confounding variables to explain some of the associations seen. In addition, the study population is from the healthcare setting which, while providing valuable information for this predominantly female cohort of workers, could limit the external validity of the results. There may also have been response bias with those experiencing difficulties with menopause more likely to complete the survey. However, given that up to 85% of women experience menopausal symptoms [7], it is likely that many of those menopausal workers who didn’t complete the survey may also be symptomatic. Although respondents provided information on treatments used, the impact of such treatments was not investigated being beyond the scope of this study.

The strengths of this study include assessing the extent of the impact of 10 specific menopausal symptoms on work rather than assessing menopausal symptoms in a general context. Symptoms that impacted employees greater than 50% of the time at work were prioritized as opposed to a symptom just merely being present while at work. In addition, the implications of symptoms on attendance, performance and career decisions were addressed. Other strengths include the inclusion of both peri-menopausal and post-menopausal women with a range of occupations and the recruitment of subjects at work rather than at menopause clinics, which may reduce potential negative bias about menopause [29].

In conclusion, this study demonstrates that menopausal employees are significantly negatively impacted by symptoms at work, by psychological and neurocognitive symptoms which affected work performance, attendance and career decisions. Many female workers will experience menopause with symptoms typically lasting for 4–8 years and in this study, three-quarters of respondents reported ongoing symptoms. It is, therefore, reasonable to conclude that, at any one time, a significant proportion of the workforce may be experiencing debilitating menopausal symptoms with potential implications for organizational service delivery. Results indicate that employees may feel compelled to reduce hours or change roles or duties, all of which may have lasting career implications for a cohort who are often highly skilled and at the peak of their careers [30]. Further research investigating the impact of menopausal symptoms at work and the benefits of available treatments and workplace accommodations is recommended to improve support for menopausal workers and to promote their occupational well-being.

## Supplementary Material

kqad078_suppl_Supplementary_Table_S1
