# Supplementary material for: Impact of menopausal symptoms on work and careers: a cross-sectional study
Source: Occup Med (Lond). 2023 Aug 5;73(6):332–8. doi: 10.1093/occmed/kqad078 (PMC10540666; doi:10.1093/occmed/kqad078)
Supplement: kqad078_suppl_Supplementary_Table_S1 [file kqad078_suppl_supplementary_table_s1.doc]

| **Symptom** | **Hot Flushes** (*n* = 386) | | | **Night sweats** (*n* = 387) | | | **Difficulty Sleeping** (*n* = 395) | | | **Fatigue** (*n* = 397) | | | **Poor Concentration** (*n* = 390) | | |
| --- | --- | --- | --- | --- | --- | --- | --- | --- | --- | --- | --- | --- | --- | --- | --- |
| **Impact at work (% time)** | **0-50%** | **50-100%** | ***P*** | **0-50%** | **50-100%** | ***P*** | **0-50%** | **50-100%** | ***P*** | **0-50%** | **50-100%** | ***P*** | **0-50%** | **50-100%** | ***P*** |
|  | (*n* = 292) | (*n* = 94) |  | (*n* = 303) | (*n* = 84) |  | (*n* = 208) | (*n* = 187) |  | (*n* = 184) | (*n* = 213) |  | (*n* = 218) | (*n* = 172) |  |
| Work performance | 175 (60) | 76 (81) | *******a | 187 (62) | 65 (77) | ******a | 109 (52) | 148 (79) | *******a | 92 (50) | 166 (78) | *******a | 106 (49) | 152 (88) | *******a |
| Career decisions | 91 (31) | 44 (47) | ******a | 90 (30) | 47 (56) | *******a | 55 (26) | 85 (45) | *******a | 45 (24) | 96 (45) | *******a | 58 (27) | 80 (47) | *******a |
| Sick leave | 48 (16) | 25 (27) | *****a | 52 (17) | 20 (24) | 0.166^a^ | 27 (13) | 46 (25) | ******a | 23 (13) | 50 (23) | ******a | 29 (13) | 44 (26) | ******a |
| Left a job | 4 (1) | 3 (3) | 0.368^b^ | 4 (1) | 3 (4) | 0.177^b^ | 1 (1) | 6 (3) | 0.056^b^ | 1 (1) | 6 (3) | 0.129^b^ | 4 (2) | 3 (2) | 1.0^b^ |
| Reduced hours | 24 (8) | 10 (11) | 0.472^a^ | 24 (8) | 10 (12) | 0.254^a^ | 15 (7) | 18 (10) | 0.387^a^ | 11 (6) | 23 (11) | 0.087^a^ | 15 (7) | 19 (11) | 0.148^a^ |
| Change role/duties | 16 (5) | 11 (12) | *****a | 16 (5) | 11 (13) | *****a | 12 (6) | 16 (9) | 0.281^a^ | 8 (4) | 20 (9) | *****a | 12 (6) | 16 (9) | 0.149^a^ |
| Stop night shifts | 14 (5) | 9 (10) | 0.089^a^ | 16 (5) | 7 (8) | 0.301^b^ | 10 (5) | 13 (7) | 0.364^a^ | 5 (3) | 18 (8) | *****a | 11 (5) | 12 (7) | 0.422^a^ |
| > 7 days sick leave/ year | 11 (4) | 10 (11) | *****a | 11 (4) | 10 (12) | ******b | 6 (3) | 15 (8) | *****a | 3 (2) | 18 (8) | ******a | 4 (2) | 17 (10) | *******a |

**Impact of 10 Menopausal Symptoms on Work & Careers**

**Table 1a**

| **Symptom** | **Poor Memory** (*n* = 391) | | | **Mood Changes** (*n* = 389) | | | **Increased Anxiety** (*n* = 385) | | | **Muscle/Joint Pain** (*n* = 387) | | | **Headaches** (*n* = 386) | | |
| --- | --- | --- | --- | --- | --- | --- | --- | --- | --- | --- | --- | --- | --- | --- | --- |
| **Impact at work (% time)** | **0-50%** | **50-100%** | ***P*** | **0-50%** | **50-100%** | ***P*** | **0-50%** | **50-100%** | ***P*** | **0-50%** | **50-100%** | ***P*** | **0-50%** | **50-100%** | ***P*** |
|  | (*n* = 232) | (*n* = 159) |  | (*n* = 260) | (*n* = 129) |  | (*n* = 233) | (*n* = 152) |  | (*n* = 243) | (*n* = 144) |  | (*n* = 281) | (*n* = 105) |  |
| Work  performance | 120 (52) | 141 (89) | *******a | 151 (58) | 105 (81) | *******a | 130 (56) | 123 (81) | *******a | 143 (59) | 110 (76) | *******a | 174 (62) | 80 (76) | ******a |
| Career decisions | 64 (28) | 75 (47) | *******a | 79 (30) | 59 (46) | ******a | 70 (30) | 66 (43) | ******a | 72 (30) | 66 (46) | ******a | 95 (34) | 46 (44) | 0.069^a^ |
| Sick leave | 31 (13) | 41 (26) | ******a | 36 (14) | 36 (28) | ******a | 33 (14) | 40 (26) | ******a | 33 (14) | 40 (28) | ******a | 38 (14) | 35 (33) | *******a |
| Left a job | 6 (3) | 1 (1) | 0.249^b^ | 4 (2) | 3 (2) | 0.69^b^ | 2 (1) | 5 (3) | 0.118^b^ | 3 (1) | 4 (3) | 0.432^b^ | 4 (1) | 3 (3) | 0.396^b^ |
| Reduced hours | 16 (7) | 18 (11) | 0.127^a^ | 17 (7) | 17 (13) | *****a | 14 (6) | 19 (13) | *****a | 19 (8) | 15 (10) | 0.383^a^ | 20 (7) | 14 (13) | 0.055^a^ |
| Changed role/duties | 15 (6) | 13 (8) | 0.519^a^ | 17 (7) | 10 (8) | 0.658^a^ | 13 (6) | 14 (9) | 0.173^a^ | 10 (4) | 16 (11) | ******a | 22 (8) | 6 (6) | 0.476^a^ |
| Stop night shifts | 13 (6) | 10 (6) | 0.777^a^ | 14 (5) | 9 (7) | 0.531^a^ | 12 (5) | 11 (7) | 0.398^a^ | 8 (3) | 15 (10) | ******a | 18 (6) | 5 (5) | 0.544^a^ |
| > 7 days sick leave/ year | 5 (2) | 16 (10) | *******a | 5 (2) | 16 (12) | *******a | 4 (2) | 17 (11) | *******a | 10 (4) | 11 (8) | 0.139^a^ | 12 (4) | 9 (9) | 0.097^a^ |

**Table 1b** Results are presented as *n* (%) ^a^Pearson chi-squared test, ^b^Fisher’s exact test  *****P < 0.05, **P < 0.01, *** P <0.001
